# Supplementary material for: Four Decades of Bacillus Biofertilizers: Advances and Future Prospects in Agriculture
Source: Microorganisms. 2025 Jan 17;13(1):187. doi: 10.3390/microorganisms13010187 (PMC11767708; doi:10.3390/microorganisms13010187)
Supplement: Supplementary file 1 [file microorganisms-13-00187-s001.zip › microorganisms-3427228-supplementary.pdf]

Table S1

Type of papers from 1985 to 2023.

PT	Rank	TP	Percentage (%)
Article	1	2,975	91.76
Review	2	175	5.40
Proceeding Paper	3	115	3.55
Early Access	4	11	0.34
Meeting Abstract	5	2	0.06
Retracted Publication	6	2	0.06
Editorial material	7	1	0.03
Book Chapter	8	1	0.03

PT: type of papers; TP: Total publication number of papers.

Table S2

The language of the paper (1985–2023).

Language	TP	TR	Percentage (%)
English	3,193	1	98.19
Polish	18	2	0.55
Spanish	14	3	0.43
Portuguese	11	4	0.34
German	5	5	0.15
Czech	4	6	0.12
Korean	2	7	0.06
French	1	8	0.03
Italian	1	9	0.03
Japanese	1	10	0.03
Russian	1	11	0.03
Turkish	1	12	0.03

TP: Total publication number of papers; TR: The ranking of the total number of papers.

Table S3

Articles of the first 10 countries (1985–2023).

Country	TA	TR	PR
China	896	1	1429.1
India	417	2	1406.0
USA	326	3	335.9
Pakistan	193	4	216.4
Brazil	183	5	240.5
South Korea	104	6	51.7
Egypt	97	7	86.2
Saudi Arabia	93	8	38.7
Spain	93	9	83.5
Germany	92	10	124.9

TA: Total publication number of articles; TR: The ranking of the total number of articles; PR: The ranking of population (Unit: million).

Table S4

Performance of the top 20 most productive institutions.

Institution	Country	TA	TPR (%)	TR
Nanjing Agricultural University	China	113	3.80	1
Chinese Academy of Sciences	China	111	3.73	2
Ministry of Agriculture Rural Affairs	China	103	3.46	3
Egyptian Knowledge Bank	Egypt	95	3.19	4
Indian Council of Agricultural Research Icar	India	95	3.19	5
Chinese Academy of Agricultural Sciences	China	77	2.59	6
King Saud University	Saudi Arabia	49	1.65	7
University of Chinese Academy of Sciences	China	46	1.55	8
Ataturk University	Turkey	45	1.51	9
China Agricultural University	China	45	1.51	10
Empresa Brasileira De Pesquisa Agropecuaria Embrapa	Brazil	44	1.48	11
Icar Indian Agricultural Research Institute	India	44	1.48	12
United States Department of Agriculture Usda	USA	40	1.34	13
University of Agriculture Faisalabad	Pakistan	40	1.34	14
Northwest Agriculture and Forestry University	China	38	1.28	15
Nanjing Institute of Soil Science	China	37	1.24	16
Islamia University of Bahawalpur	Pakistan	36	1.21	17
Universidade Federal Rural De Pernambuco Ufrpe	Brazil	35	1.18	18
Universidade Estadual Paulista	Brazil	34	1.14	19
Comsats University Islamabad Cui	Pakistan	31	1.04	20

TA: Total publication number of articles; TPR%: Percentage of total papers published; TR: The ranking of the total number of articles.

Table S5
Top 20 authors by number of articles (1985–2023).

Authors	TA	Institution	Country	Research Field
Shen, Qirong	77	Nanjing Agricultural University	China	Soil Science, Microbial Ecology
Stamford, Newton Pereira	27	Federal University of Pernambuco	Brazil	Soil Fertility, Microbial Fertilizers
Li, Rong	22	Nanjing Agricultural University	China	Plant Pathology, Microbial Ecology
Hussain, Azhar	21	University of Haripur	Pakistan	Microbial Biotechnology, Soil Science
Saeid, Agnieszka	19	Wrocław University of Environmental and Life Sciences	Poland	Environmental Biotechnology
Sahin, F	19	Ondokuz Mayis University	Turkey	Plant Pathology
Jastrzebska, Magdalena	18	Warsaw University of Life Sciences	Poland	Environmental Science
Ahmad, Maqshoof	17	University of Agriculture Faisalabad	Pakistan	Plant Pathology
Ling, Ning	17	Nanjing Agricultural University	China	Microbial Ecology
Kostrzewska, Marta K.	17	Warsaw University of Life Sciences	Poland	Environmental Biotechnology
Turan, Metin	17	Yeditepe University	Turkey	Soil Science
Zhang, Ruifu	16	Nanjing Agricultural University	China	Microbial Ecology
Huang, Qiwei	16	Nanjing Agricultural University	China	Plant Pathology
Xu, Yangchun	15	Nanjing Agricultural University	China	Soil Science
Zhu, zhu	15	Nanjing Agricultural University	China	Plant Pathology
Santos, Carolina	14	Federal University of Pernambuco	Brazil	Soil Fertility, Microbial Fertilizers
Chen, Sanfeng	14	Nanjing Agricultural University	China	Soil Microbiology
Ruan, Yunze	14	Nanjing Agricultural University	China	Soil Microbiology
Bano, Asghari	14	Quaid-i-Azam University	Pakistan	Plant Physiology
Kloepper, Joseph W.	13	Auburn University	USA	Plant Growth-Promoting Rhizobacteria

TA: Total publication number of articles.

Tables S6

The 20 most productive journals during 1985–2023.

Journal	Country	TA	IF (2023)	CiteScore
Frontiers In Microbiology	Switzerland	99	4.0	7.8
Agronomy-Basel	Switzerland	86	3.949	3.6
Microorganisms	Switzerland	64	4.926	5.4
Applied Soil Ecology	Netherlands	51	4.552	6.2
Scientia Horticulturae	Netherlands	46	2.946	3.2
Frontiers in Plant Science	Switzerland	45	6.627	7.3
Microbiological Research	Germany	43	5.072	6.1
Science of the Total Environment	Netherlands	43	10.753	12.1
Plos One	USA	42	3.752	4.3
Bioresource Technology	UK	41	9.700	20.8
Scientific Reports	UK	40	3.800	7.7
Biology And Fertility of Soils	Germany	39	4.638	6.4
Plants-Basel	Switzerland	37	4.658	4.9
Plant And Soil	Netherlands	36	4.336	5.7
Soil Biology & Biochemistry	UK	34	6.030	8.0
Journal of Soil Science and Plant Nutrition	Chile	33	2.247	2.7
Communications soil science and plant analysis	USA	31	1.300	3.3
Environmental Science and Pollution Research	Germany	31	4.223	6.6
Journal of plant nutrition	USA	31	1.600	4.4
Journal of applied microbiology	UK	30	3.774	5.1

IF：Impact Factor; TA: Total publication number of articles; AC: Average number of citations.

Table S7

The top 10 research areas in terms of number of articles (1985–2023).

Research areas	TA	TR	Percentage (%)
Agriculture	999	1	33.58
Environmental Sciences Ecology	593	2	19.93
Microbiology	564	3	18.96
Plant Sciences	547	4	18.39
Biotechnology Applied Microbiology	412	5	13.85
Science Technology Other Topics	209	6	7.03
Engineering	144	7	4.84
Chemistry	137	8	4.61
Biochemistry Molecular Biology	96	9	3.23
Entomology	77	10	2.59

TA: Total publication number of articles; TR: The ranking of the total number of articles.

Table S8

Top 42 keywords with the highest number of occurrences (1985–2023).

Keywords	Number	TR	Percentage (%)	TLS
PGPR	393	1	6.09	927
Biofertilizer	361	2	5.59	824
Plant growth	286	3	4.43	665
Bacillus	237	4	3.67	579
Phosphorus	281	5	3.38	500
Microbial community	200	6	3.10	346
Biolog control	122	7	1.89	292
Bacillus subtilis	108	8	1.67	237
Rhizosphere	106	9	1.64	270
Endophyte	98	10	1.52	221
Compost	97	11	1.50	192
Fertilizer	95	12	1.47	206
Maize	73	13	1.13	210
Pseudomonas	71	14	1.10	225
Yield	69	15	1.07	171
Bioinoculant	68	16	1.05	154
16S rRNA	67	17	1.04	139
Rhizobacteria	63	18	0.98	162
Bacteria	62	19	0.96	145
Inoculant	60	20	0.93	128
IAA	57	21	0.88	161
Antibiotic	55	22	0.85	107
Nitrogen fixation	54	23	0.84	147
Salinity stress	54	24	0.84	134
Wheat	52	25	0.81	139
Bacillus megaterium	51	26	0.79	119
Sustainable	51	27	0.79	140
Nutrient uptake	50	28	0.77	126
Biochar	49	29	0.76	105
AMF	42	30	0.65	95
Fusarium wilt	42	31	0.65	107
High-throughput sequencing	42	32	0.65	85
Rice	41	33	0.64	98
Bacillus amyloliquefaciens	36	34	0.56	93
Paenibacillus	35	35	0.54	89
Enzyme	34	36	0.53	81
Bioremediation	33	37	0.51	47
Nitrogen	33	38	0.51	89
Soil	32	39	0.50	71
Tomato	32	40	0.50	97
potassium	30	41	0.46	69

siderophore	30	42	0.46	81

TR: The ranking by keyword occurrences; TLS: Total link strength of the keyword.

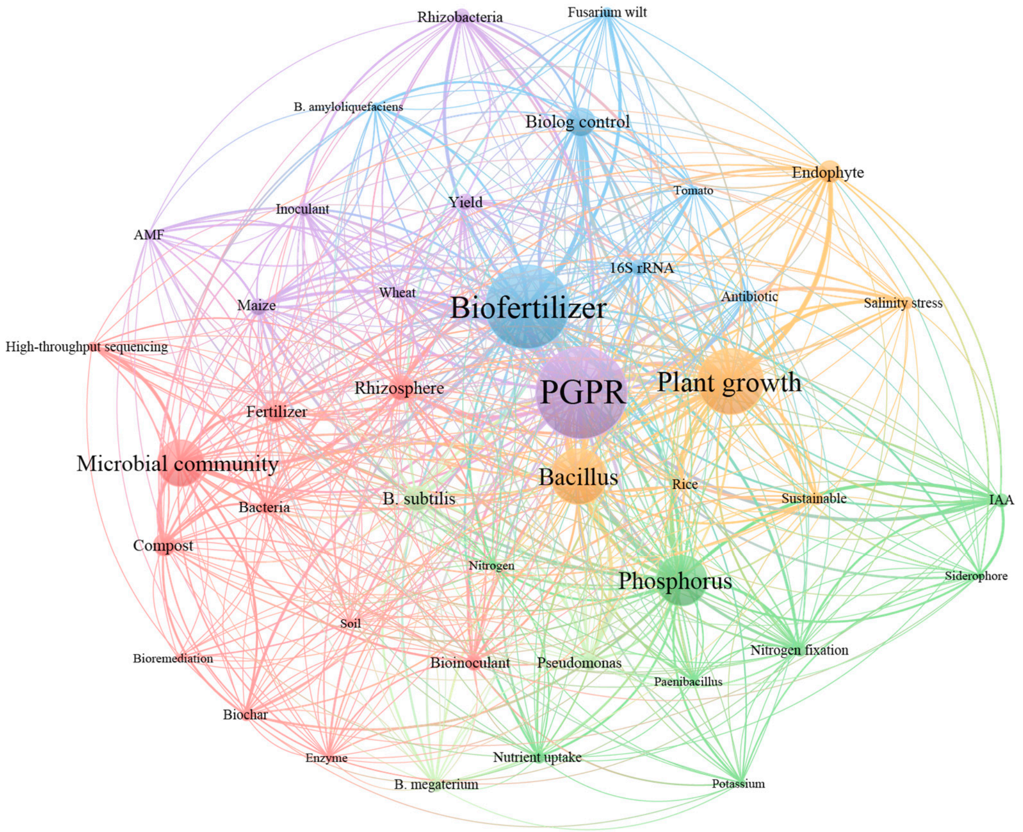

Figure S1. Co-occurrences network of the top 42 most frequently occurring keywords from 1985 to 2023.

Larger nodes indicate higher frequency of keyword occurrence, while wider edges represent a greater number of co-occurrences between keywords.
